# VSCode-Antimony: a source editor for building, analyzing, and translating antimony models

**DOI:** 10.1093/bioinformatics/btad753

**Published:** 2023-12-14

**Authors:** Steve Ma, Longxuan Fan, Sai Anish Konanki, Eva Liu, John H Gennari, Lucian P Smith, Joseph L Hellerstein, Herbert M Sauro

**Affiliations:** NVIDIA Corporation, Redmond, WA 98052, United States; Department of Mathematics, University of Washington, Seattle, WA 98195, United States; Allen School of Computer Science, University of Washington, Seattle, WA 98195, United States; Allen School of Computer Science, University of Washington, Seattle, WA 98195, United States; Biomedical and Health Informatics, University of Washington, Seattle, WA 98195, United States; Department of Bioengineering, University of Washington, Seattle, WA 98195, United States; eScience Institute, University of Washington, Seattle, WA 98195, United States; Department of Bioengineering, University of Washington, Seattle, WA 98195, United States

## Abstract

**Motivation:**

Developing biochemical models in systems biology is a complex, knowledge-intensive activity. Some modelers (especially novices) benefit from model development tools with a graphical user interface. However, as with the development of complex software, text-based representations of models provide many benefits for advanced model development. At present, the tools for text-based model development are limited, typically just a textual editor that provides features such as copy, paste, find, and replace. Since these tools are not “model aware,” they do not provide features for: (i) model **b**uilding such as autocompletion of species names; (ii) model **a**nalysis such as hover messages that provide information about chemical species; and (iii) model **t**ranslation to convert between model representations. We refer to these as BAT features.

**Results:**

We present VSCode-Antimony, a tool for building, analyzing, and translating models written in the Antimony modeling language, a human readable representation of Systems Biology Markup Language (SBML) models. VSCode-Antimony is a source editor, a tool with language-aware features. For example, there is autocompletion of variable names to assist with model building, hover messages that aid in model analysis, and translation between XML and Antimony representations of SBML models. These features result from making VSCode-Antimony model-aware by incorporating several sophisticated capabilities: analysis of the Antimony grammar (e.g. to identify model symbols and their types); a query system for accessing knowledge sources for chemical species and reactions; and automatic conversion between different model representations (e.g. between Antimony and SBML).

**Availability and implementation:**

VSCode-Antimony is available as an open source extension in the VSCode Marketplace https://marketplace.visualstudio.com/items?itemName=stevem.vscode-antimony. Source code can be found at https://github.com/sys-bio/vscode-antimony.

## 1 Introduction

Developing credible biochemical models in systems biology can be invaluable for creating novel medical diagnostics, commercially viable metabolic pathways as well as help drive basic research in cell biology. Although modeling is time-consuming and knowledge-intensive, there are many approaches to mitigating these challenges. One approach is to employ a graphical user interface (GUI) to guide users and reduce errors in model building (Hoops *et al.*, 2006). GUI systems are particularly helpful for less experienced and novice users. However, text-based representations of models provide another approach to dealing with large-scale models (Choi *et al.*, 2018). We see the situation as being analogous to the development of complex software where text-based approaches are widely used.

There are several text-based representations of biochemical models. One popular choice is the Systems Biology Markup Language (SBML), a community standard for model exchange in an XML format (Hucka *et al.*, 2003). SBML is an effective computer-readable model representation, but it is verbose and obscure as a human readable model description language. This motivated the development of Antimony (Smith *et al.*, 2009), a human readable textual representation of SBML. For example, Antimony expresses reactions as chemical formulas, a representation that is quite familiar to developers of biochemical models.

Developing biochemical models using text is currently done using a text editor that provides features such as copy, paste, find, and replace. Examples of text editors include vim (Robbins *et al.*, 2008), emacs (Cameron *et al.*, 2005), Windows notepad, and sublime (Peleg, 2013). Text editors make it easy to change the names of model variables. For example, we can change all occurrences of the names of the chemical species AMp and cAMp to AMP and cAMP by doing a replace of AMp with AMP. A text editor can also accelerate model building. To illustrate this, consider the chemotaxis model in BioModels model 200 (Bray and Bourret, 1995). The model contains several phosphorylation reactions of the form X => Xp, where X is one of the molecules: WAA, WWAA, TTAA, TTWAA. Rather than entering a phosphorylation reaction for each molecule, we can accelerate model building by placing the string “X => Xp” into the editor “clipboard” and repeating the following for each molecule: (i) paste the copied reaction and (ii) replace all occurrences of X with the molecule to be phosphorylated. A final appeal of a text editor is that many text editors are programmable, and so it possible to create automation in support of model building and analysis.

Despite these benefits, developing biochemical models with a text editor has serious shortcomings. Foremost, *text editors are not “model aware*.*”* That is, text editors do not know about species, reactions, compartments, or parameters. Also, text editors do not know the syntax of model statements. This severely limits the ability of text editors to support model **b**uilding (e.g. autocompletion of variable names), model **a**nalysis (e.g. detecting errors in model statements), and model **t**ranslation (e.g. converting between SBML and Antimony or other formats). We use the term *BAT* feature to refer to capabilities that support model building, analysis, and translation.

The absence of BAT features is analogous to problems faced in the early days of software engineering. At that time, software engineers used text editors that lacked knowledge of their programming language. In recent years, there has been a dramatic increase in the productivity and quality of software engineering as a result of source editors, Source editors are text editors with knowledge of the programming language. This knowledge enables source editors to provide syntax highlighting, code navigation (e.g. displaying method signatures), and error detection.

Our focus is on SBML models. Since SBML is a community standard, one approach is to create a source editor for SBML, as is done in Rodriguez *et al.* (2007). This system provides syntax highlighting of XML and error checking of XML syntax. Although these capabilities are useful at times, we believe that working at the level of XML is too low for most model developers who are thinking in terms of chemical reactions not in terms of XML angle brackets.

This article describes a source editor for the Antimony modeling language. We chose Antimony because it provides a good match with the conceptual level at which models are developed. For example, the reaction $O_{2}+2H_{2}\to 2H_{2}O$ is written in Antimony as O2 + 2 H2 -> 2 H2O for the chemical species O2, H2, H2O. A further benefit of using Antimony is that it supports model hierarchies through nesting of models. Such hierarchical representations can provide considerable benefits for scaling and model reuse. Our system, VSCode-Antimony, implements BAT features for building, analyzing, and translating Antimony models.

## 2 Methods and materials

Building a source editor for the Antimony language requires capabilities beyond those that are provided by a text editor. A text editor provides features such as copy, paste, find, and replace. However, a source editor knows about the modeling language itself. Examples include knowing the type of a symbol (e.g. species, parameter), changing the visual characteristics of symbols and statements (e.g. highlighting, underlining), accessing model related knowledge sources (e.g. ChEBI; Degtyarenko *et al.*, 2007), and translating between model representations.

Although Antimony is a language for modeling biochemical reactions, it is also a computer language. As such, Antimony has a formal grammar just as there are formal grammars for as python, Java, and C. This observation led us to build the Antimony source editor as an extension to an existing source editor for computer programming. We chose VSCode (Garcia and Rojas, 2022) because it is widely used, open source, and has a well-supported application programming interface (API) for implementing extensions. The API for VSCode extensions provides many features we use in VSCode-Antimony: hover information (providing information when hovering over a symbol), autocompletion (completing partially entered text), jump to definition (navigating to related information about a symbol), error checking the structure of statements, highlighting, and underlining to indicate errors and warnings.

The most significant consideration in implementing a VSCode extension is describing the grammar of the language for which the source editor is intended. VSCode provides a convenient way to describe the language grammar in terms of python regular expressions. However, parts of the Antimony language have a complex grammar. For example, an Antimony event specifies actions taken when a condition arises as determined by a Boolean expression of model variables. This can result in a fairly complicated statement that is challenging to parse correctly.

Although VSCode provides a rich API for writing extensions, it is not sufficient for implementing a source editor for a biochemical modeling language. For example, VSCode-Antimony provides features for translating between representations of biochemical models, such as translating between Antimony and SBML. Although it is common in software to do one-way translations (e.g. compilation), two way translations are rare, especially maintaining two different representations of the same information with bi-directional editing.

There is yet another requirement for developing biochemical models that differs from software engineering. Unlike programming languages, modeling languages often need to relate symbols used in the model to external “knowledge sources.” This linkage is essential to understanding the assumptions and scope of a model, both to interpret model results and to use the model as a building block in larger models. Example knowledge sources include ChEBI (Chemical Entities of Biological Interest), which describes small molecules, Rhea (Bansal *et al.*, 2022), which describe reactions, and the Gene Ontology (Gene Ontology Consortium, 2004), which provides descriptions of pathways and biological processes. Annotations provide the connection between symbols and knowledge sources. Often there are many choices for a seemingly simple molecule. For example, ChEBI lists approximately 1000 choices for “glucose.” VSCode-Antimony has capabilities for querying knowledge sources to assist modelers with annotating their models.

## 3 Results

This section describes how the capabilities detailed in Section 2 are used to construct the BAT features supported by VSCode-Antimony. Section 3.1 lists the BAT features. Subsequent sections demonstrate how the features support model augmentation (Section 3.3), analysis, and debugging (Section 3.4).

### 3.1 BAT features

VSCode-Antimony provides a rich set of BAT features that build on the capabilities described in Section 2. Table 1 provides a summary.

**Table 1.** BAT features in VSCode Antimony.

BAT category	VSCode-Antimony feature
Build	Editing SBML files as Antimony
	Browsing BioModels
	Autocompletion
	Model import
	Automatic rate law insertion
	Annotation creation
Analyze	Syntax highlighting
	Annotation highlights
	Hover messages
	Model navigation
	Error and warning detection
Translate	Bidirectional editing
	SaveAsAntimony
	SaveAsSBML

#### 3.1.1 Build

Model building is about creating a new model and/or modifying an existing model.

**Figure 1.**
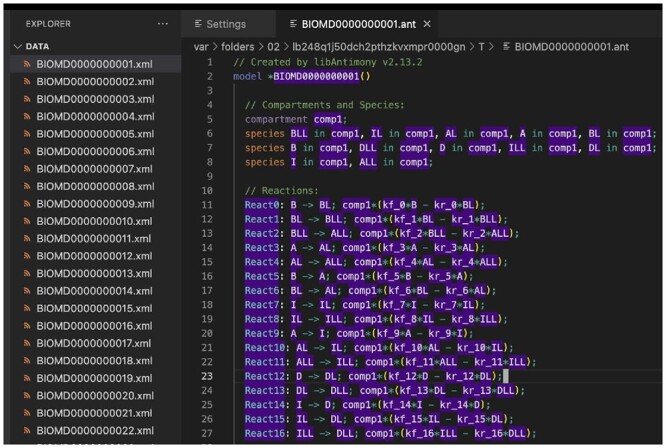
Editing an SBML file as Antimony. Clicking on BIOMD0000000001.xml causes the XML to be converted to antimony and opens a temporary file. When the Antimony file is saved, it is converted to an SBML file and replaces the original XML file.

**Editing SBML files as Antimony** allows modelers to browse and update an SBML file (in XML) as a human readable Antimony file. As displayed in Fig. 1, when the user clicks on BIOMD0000000001.xml, VSCode-Antimony automatically converts the XML into Antimony, and creates a temporary file with the same name but with the ant extension. A notification appears indicating that the XML file is being edited as an Antimony file. Changes made to the ant file are propagated back to the original XML file.

**Figure 2.**
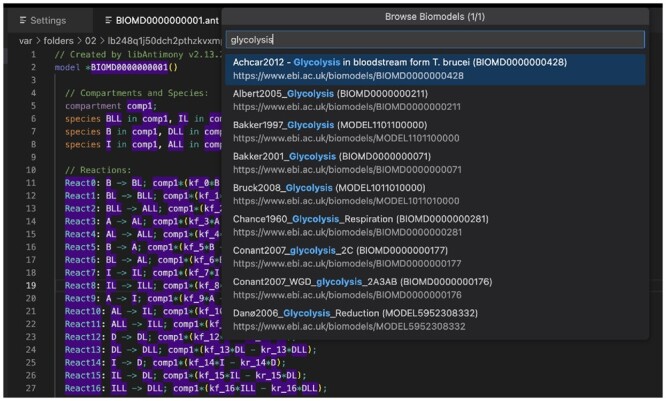
Browsing BioModels. Users can search BioModels, and view a selected model in the Antimony representation instead of XML.

**Browsing BioModels** provides modelers with a simple way to access models in BioModels in a human readable format. The “Browse Biomodels” option can be accessed when the user right clicks. As illustrated in Fig. 2, the user is presented with a text box. Entering an integer into the text box causes VSCode-Antimony to search for that model number. Entering a string produces a list of models with that string. When the user selects a model in the list, the model is downloaded, converted to Antimony, and displayed in the editor.

**Autocompletion** is extremely valuable for model building. This feature operates while the modeler is typing. For example, suppose glucose_rate is a parameter name in the model and the user types “glu”, then the system suggests names that begin with “glu”. Since VSCode-Antimony has knowledge of the Antimony grammar, suggestions are context dependent. For example, the parameter glucose_k1 is not suggested as a reactant or a product; however, it could be suggested while typing a rate law.

**Figure 3.**
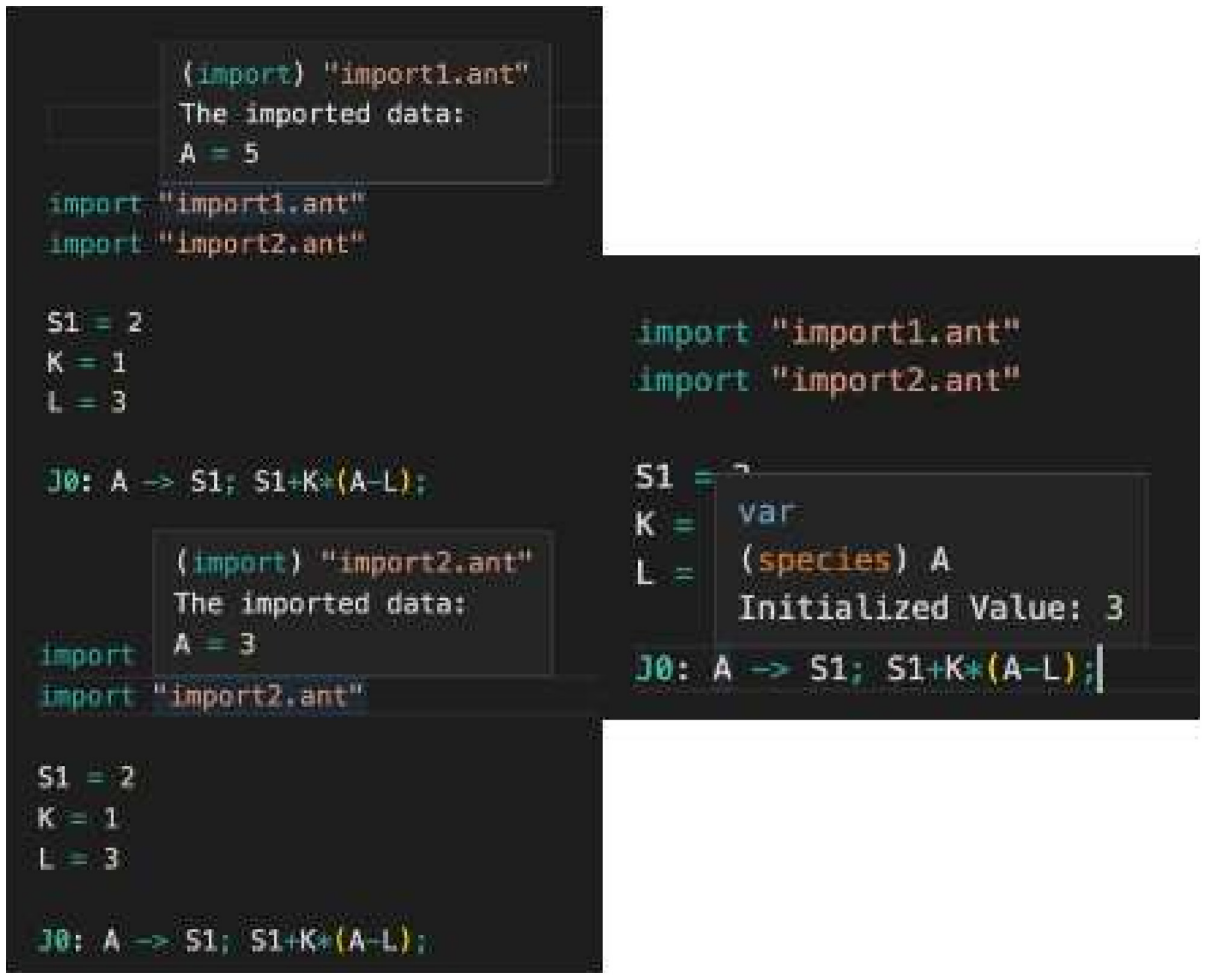
Illustration of the hover preview provided by the import feature.

**Model import** allows modelers to reuse models developed elsewhere. Reuse has been essential to the rapid growth in size and complexity of software. By importing an Antimony file, the modeler has access to the model elements in that file, such as floating species, reactions, parameters, and function definitions (e.g. for rate laws). As illustrated in Fig. 3, hovering over an import statement brings up a preview of what is being imported.

**Automatic rate law insertion** helps modelers with selecting and instantiating a rate law for a reaction. Types of rates laws include irreversible mass action, Michaelis–Menten, and Hill expressions. Once the rate law type is known, the rate law must be instantiated for the reaction. VSCode facilitates selecting the rate law type, and it automates rate law instantiation.

**Figure 4.**
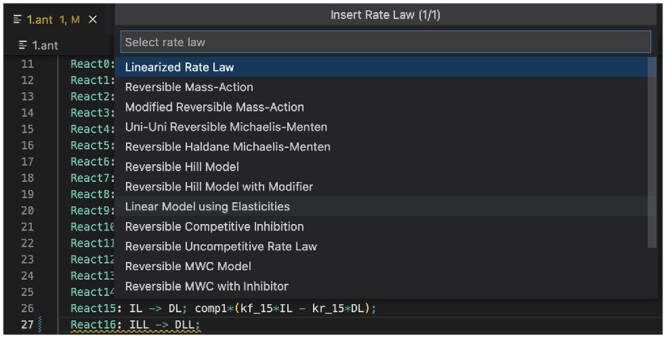
Example of rate law insertion.

Figure 4 displays an example of rate law insertion for the reaction React1: BLL + DL => ILL. The user right-clicks and selects “Insert Rate Law.” Then a drop-down menu appears that allows for selection of the rate law functional form. In the figure, “Irreversible Mass-Action Bi-Uni” is chosen. VSCode-Antimony then provides for in-line entry of required constants to instantiate the rate law.

**Figure 5.**
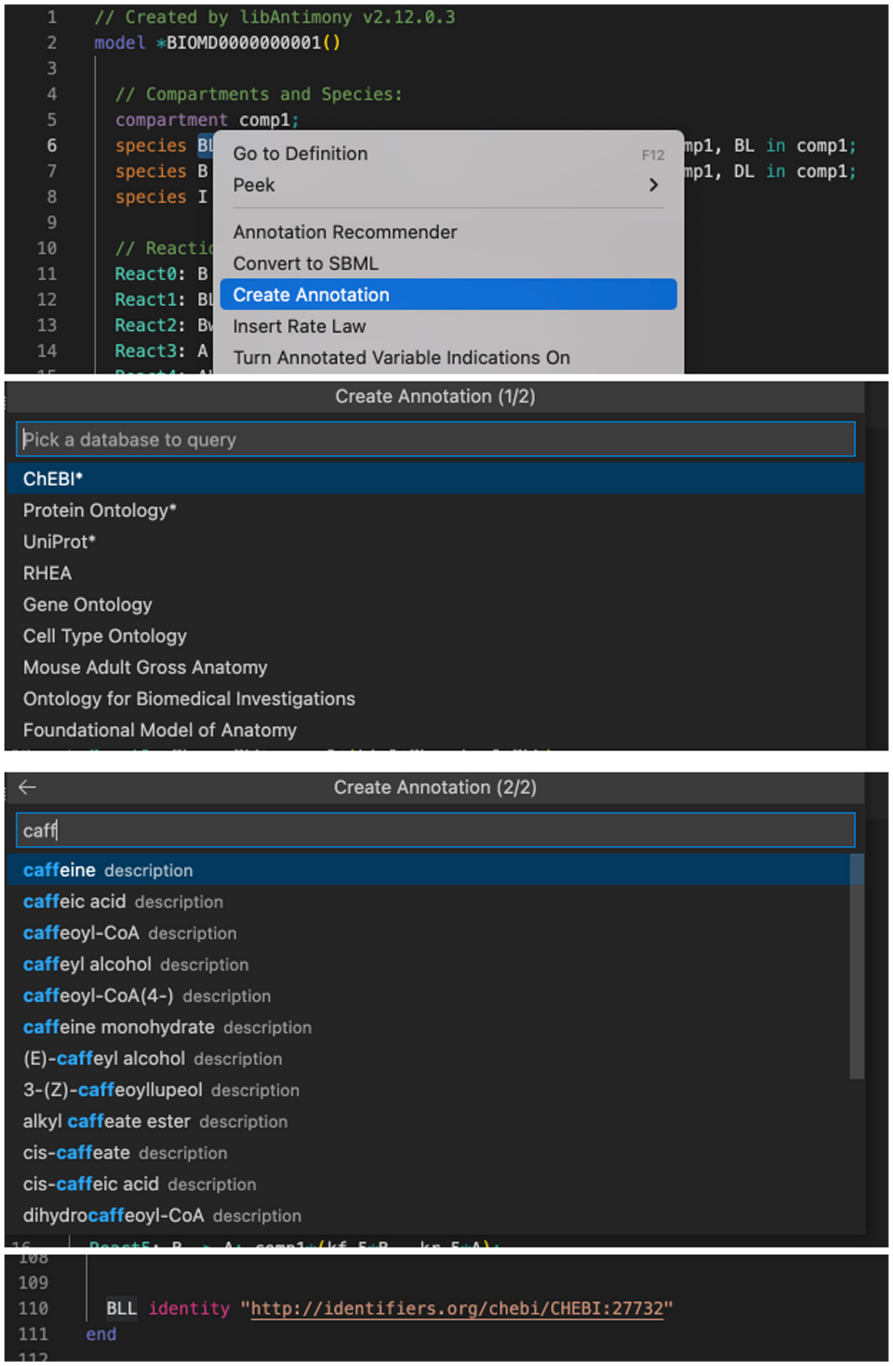
Annotation creation steps: (1) The user selects a model element to annotate, and does a right click. (2) VSCode-Antimony displays a pop-up menu. The user selects “Create Annotation” (3) VSCode-Antimony displays a list of knowledge sources from which annotations may be selected. Knowledge sources with an asterisk (“*”) are type appropriate. (4) The user enters a descriptive text for the model element being annotated. VSCode-Antimony displays query responses. (5) The user selects an annotation, and an annotation statement is entered at the bottom of the file.

**Annotation creation** simplifies the creation of annotations for model elements such as chemical species, reactions, and compartments. This feature leverages the built-in capability of VSCode-Antimony to query knowledge sources that contain annotations such as ChEBI for chemical species and Rhea for reactions. The feature leverages the grammar processing capabilities of VSCode-Antimony to determine the type of the model element, and from this, select appropriate knowledge resources for that type. By type appropriate, we mean that if the symbol being annotated is a chemical species, then we display first knowledge sources for chemical species (e.g. ChEBI). On the other hand, if what is being highlighted is a reaction, we display first knowledge sources for reactions (e.g. Rhea). Type appropriate knowledge sources are indicated by an asterisk (“*”). The user experience is depicted in Fig. 5.

#### 3.1.2 Analyze

Model analysis is about understanding the elements of the models (e.g. species, reactions, compartments), and how they interact over time.

**Figure 6.**
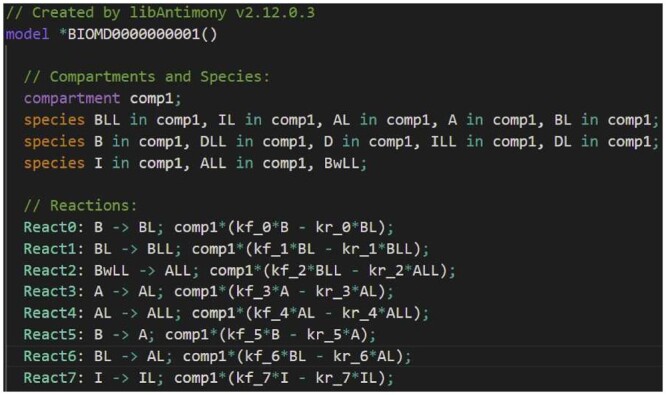
An Antimony model (Edelstein *et al.*, 1996) highlighted by VSCode-Antimony.

**Syntax highlighting**, also known as syntax coloring, is a feature offered by many source editors for programming languages to display different colors according to the type of language element. A study published at the PPIG conference (Sarkar, 2015) suggests that syntax highlighting can significantly reduce the time taken for a programmer to internalize the semantics of a program. We believe that similar benefits are possible for modelers. VSCode-Antimony highlights reaction names, species names, operators, event names, units, functions, and more. Figure 6 displays an example of VSCode-Antimony syntax highlighting.

**Annotation highlighting** provides a fast, visual indication of the model elements that are annotated or are *not* annotated (depending on a global setting accessible through a right click). This feature facilitates analysis by communicating the modeling elements for which more detailed information is available. It also provides a way to identify model elements that should be annotated.

**Figure 7.**
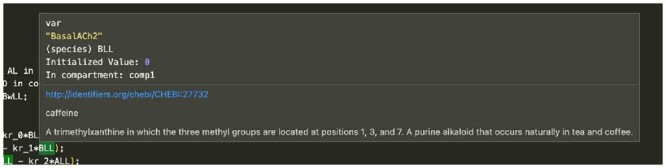
Example of hover messages for a species.

**Hover messages** are text displayed on a mouse-over. In software source editors, mouse-over is widely used to display the type and values of variables and the types of arguments to functions. Providing this information in context reduces searching through files. Users of VSCode-Antimony see hover messages for a wide variety of model elements; the message content depends on the type of the model element. For variables, (e.g. species: parameter, compartment), hover displays the variable type, assigned value, annotation information. For functions, hover displays the types of the arguments to functions. And for reactions, hover displays annotation information. Figure 7 displays a hover message for the chemical species BLL in a model.

**Figure 8.**
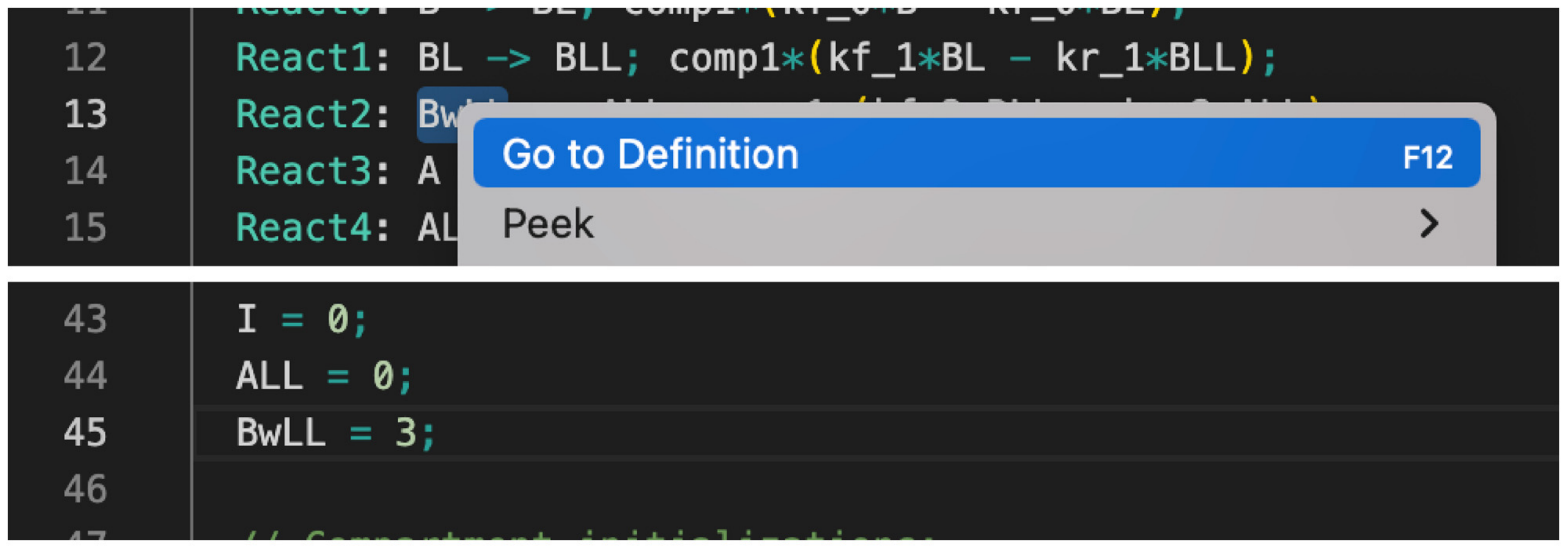
Example of code navigation.

**Model navigation** is a “model aware” way of browsing a model file. The analogous feature in software source editors is called “code navigation,” and provides a way to view the definitions of variables and functions. In VSCode-Antimony, navigation is provided to the definition of model symbols and the initialization of variable values. This is done by (a) selecting a model symbol, (b) right-clicking, and (c) selecting “Go to Definition.” Figure 8 illustrates how this works.

**Error and warning detection** is widely used in software source editors to increase the quality and productivity of software engineering. Source editors can detect *static* errors, errors that can be detected without running code. Examples include syntax errors and flow control issues, such as code that is unreachable (e.g. code that it is preceded by a return statement). In VSCode, errors and warnings are indicated by a “squiggly underline” beneath the questionable text.

There are many examples of static testing in Systems Biology. MEMOTE (Lieven *et al.*, 2020) generates a report on annotation quality, and MASSpy (Haiman *et al.*, 2021) identifies missing values, parameters, fluxes, and concentrations for Systems Biology models. At present, VSCode-Antimony provides a subset of these features by detecting: syntax errors (based on grammar analysis of the Antimony Language), uninitialized variables, and type mismatches between the arguments of a function call and its definition. We distinguish between errors and warnings based on the effect on running a simulation. An error means that the simulation will not run; warnings indicate a questionable modeling practice (e.g. overriding the value of a parameter). Errors are indicated by a red squiggly line, and warnings by a yellow squiggly line. Table 2 displays to errors and warnings detected by VSCode-Antimony.

**Table 2.** Errors (E) and warnings (W) in VSCode-Antimony. Element types are species (Spc), parameters (Prm), compartments (Cpt), reactions (Rea), rate laws (Rl), function (Fun), modular models (Mdl), and general (Gen).

Type	Issue	E/W
Spc	No initial value	W
Spc	Overriding previous value assignment	W
Prm	No initial value	E
Prm	Overriding previous value assignment	W
Cpt	No initial value	W
Cpt	Overriding previous value assignment	W
Rea	Referencing uninitialized species	W
Rea	Invalid arithmetic expression	W
Rl	Variable is not a fixed-species in reaction	E
Rl	Overriding rate rule	W
Evt	Overriding defined event trigger	W
Fun	Unused parameter	W
Fun	Calling undefined function	E
Fun	Incorrect function parameters	E
Fun	Incorrect/incompatible parameter type	E
Fun	Defining a new function with a used name	E
Mdl	Calling undefined modular model	E
Mdl	Incorrect modular model parameters	E
Mdl	Incorrect parameter type	E
Gen	Overriding display name	W
Gen	Overriding incompatible type to previous type	E
Gen	Unexpected token	E
Gen	Unexpected newline or EOF	E

#### 3.1.3 Translate

Model developers sometimes need to consider more than one representation of a model. Model translation converts between model representations. At present, VSCode-Antimony has features for converting between Antimony and SBML/XML. Other model representations can be of interest as well. For example, CellML represents a biochemical model as a system of differential equations.

**Bidirectional editing** provides a way to simultaneously edit two representations of the same model. This is useful if there are imperfections in the translation between model representations. For example, at present, there is no way to represent comments in SBML, and so comments in an Antimony file are lost when the file is translated to SBML. Another reason for bidirectional editing is to support tool developers who generate new model representations.

**Figure 9.**
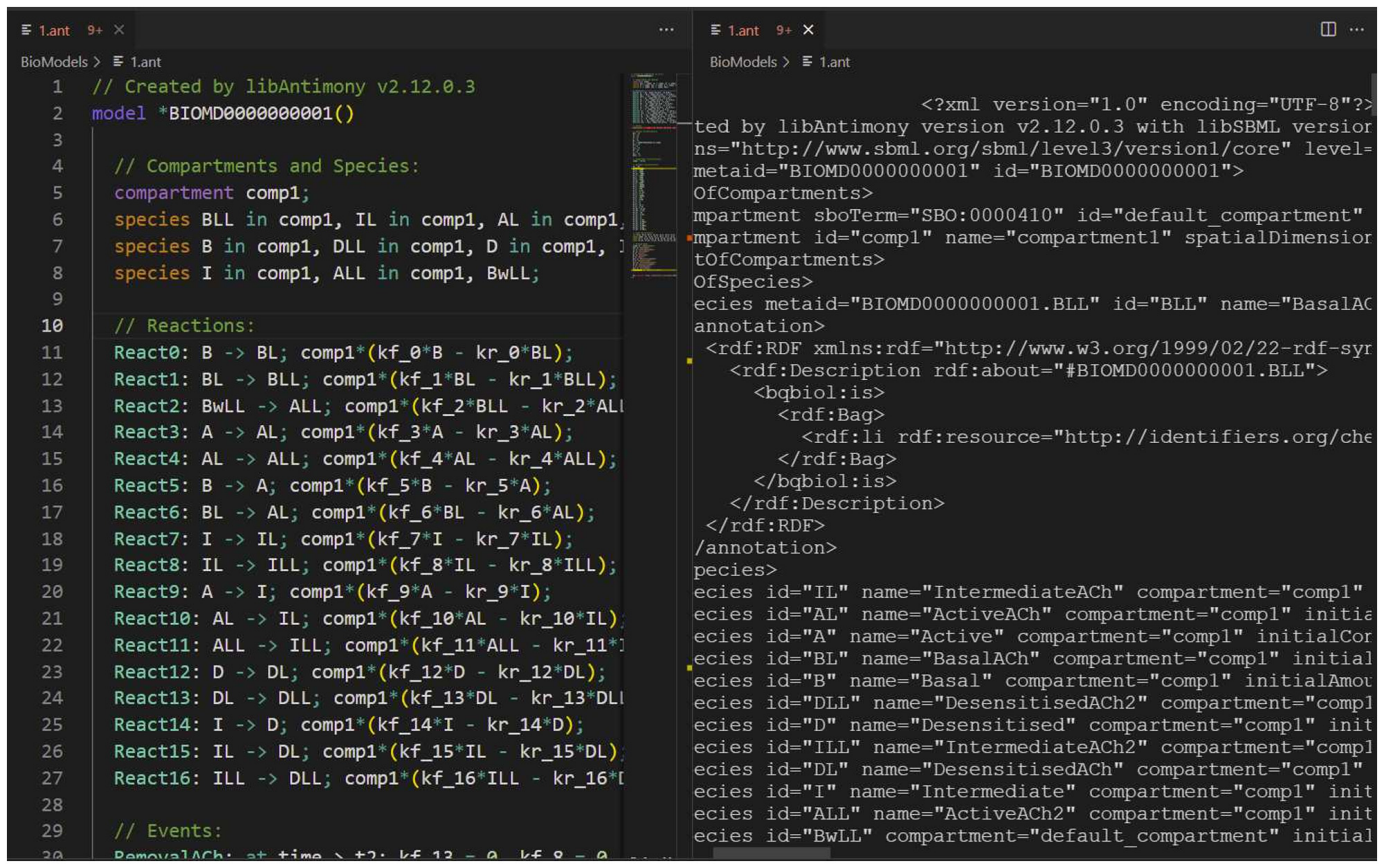
Bidirectional editing of Antimony and SBML representations of a model.

VSCode-Antimony displays model representations in a split-screen mode, as illustrated in Fig. 9. Changes made in one model representation are propagated to the other representation.

**SaveAs features** provide for translating the current model representation. VSCode-Antimony provides for saving an Antimony file as SBML, and for saving an SBML file as Antimony. This feature is accessed by a right-click and selecting “Convert to Antimony” or “Convert to SBML.”

### 3.2 Installation, documentation, testing, limitations

Before installing VSCode-Antimony, you must first install VSCode as described in https://code.visualstudio.com/. VSCode-Antimony is a VSCode extension. Start VSCode, and then open the VSCode extensions. Search for “Antimony,” and then select “Antimony Extension Pack.” The extension pack automatically installs two extensions: the Antimony extension which provides language supports, and the Antimony Syntax which provides the syntax coloring scheme for the Antimony extension. The information displayed when the VSCode-Antimony extension is installed may include additional instructions.

The Visual Studio Marketplace provides some documentation of VSCode-Antimony; more details can be found in the project GitHub repository at https://github.com/sys-bio/vscode-antimony.

We have developed extensive unit tests using pytest, and continuous integration using GitHub actions.

VSCode-Antimony supports the analysis of deterministic kinetic models, and so does not support features related to uncertainty and statistical analysis.

### 3.3 Use case

This scenario describes a use case in which VSCode-Antimony helps with modifying an existing model. The scenario adds a reaction to BioModels 200, a model of chemotaxis with 32 reactions (Bray and Bourret, 1995).


Yp+TTWWAAp=>Ypp+TTWWAA

We want the rate law for this reaction to be reversible mass action:


cell*phosphotransfer_r12_k1*Yp*TTWWAAp

The first step is to get the existing model into an environment where it can be modified. Using a text editor requires: (a) browsing to BioModels; (b) downloading the SBML file for the model; and (c) converting the SBML file into a human readable representation. However, with VSCode-Antimony, we use the “Browse BioModels” feature, enter 200, and select “Bray1995.” We see the model in an Antimony representation. We save the model as a new file.

We then proceed as follows:

1. The user types
  Yp+TTWW
  At this point, the autocompletion feature provides assistance; it suggests the text
  Yp+TTWWAA
  The user presses newline to accept the text. Then, autocompletion provides a further suggestion:
  Yp+TTWWAAp
  Again, the user presses newline to accept. There is a similar user experience when typing the right-hand-side of the reaction until the following text has been entered:
  Yp+TTWWAAp=>Ypp+TTWWAA
2. To specify reaction kinetics, the user employs automatic rate law insertion. The user selects the rate law “irreversible mass action,” and current line appears as:
  Yp+TTWWAAp=>Ypp+TTWWAA; __*Yp*TTWWAAp
  The underscore indicates that the user needs to supply text, in this case a parameter name that is a rate constant. The user types the necessary text, and presses the return key. The reaction has been entered.
3. The user right clicks on the reaction, and selects “Create Annotation” to create an annotation for the reaction.

### 3.4 Debugging a model

Next, we consider a use case for analyzing an Antimony model. The particulars are as follows. The modeler has completed entering the model, and has done a simulation. The output of the simulation shows that Y is far too small and Yp is far too large. This suggests that one of the following is true for Y:

- H1a: Y is degraded too rapidly.
- H1b: Y is not synthesized fast enough.

And, one of the following is true for Yp:

- H2a: Yp is synthesized too rapidly.
- H2b: Yp is not degraded enough.

So, there are four cases to investigate. The first is that both H1a and H2a are true. We denote this by (H1a, H2a). The other cases are (H1a, H2b), and (H1b, H2a), (H1b, H2b).

We illustrate how to investigate (H1a, H2a).

1. We search for all reactions in which Y appears before =>, and Yp appears after =>; that is, Y is a reactant and Yp is a product. VSCode-Antimony allows using editors that search for regular expressions (e.g. vim). The modeler enters the search string “Y .*=.*Yp.” This search string looks for the occurrence of “Y” followed by a space, followed by any number of characters, followed by “=”, followed by any number of characters, and then followed by “Yp.” The search returns 7 of the 32 reactions.
2. We look for errors or warnings in these statements, as indicated by the yellow or red squiggly lines.
3. Seeing no errors, we examine each reaction in turn. The first is:
  Y=>Yp; cell*phosphorylation_r7_k1*Y
4. We do a string search to find reactions in which Yp is degraded and Y is synthesized. The search yields
  Yp=>Y; cell*phosphorylation_r8_k1*Yp
5. We use the VSCode-Antimony hover message to see if phosphorylation_r7_k1 is much larger than phosphorylation_r8_k1 since this would explain the data that we observe.
6. The process continues with the remaining six reactions returned in the search in step (1).

## 4 Discussion

Our interest in text-based model development follows from the observation that experienced software engineers use text-based tools to develop complex software. This observation led us to build VSCode-Antimony, a source editor for developing biochemical models using the Antimony modeling language.

Modern software engineering relies heavily on Interactive Development Environments (IDEs) such as PyCharm, Visual Studio, and VSCode. These tools provide much more than source editing. They also provide an environment for running, debugging, and collaborative development.

We believe that the development of biochemical models likely has requirements similar to those for developing complex software. For example, we expect that a biochemical IDE could run simulations and view the results. Results should be accessible either by a hover over a variable or through a separate results pane (as RStudio) (van der Lo and de Jorge, 2023).

There are ways in which we expect that a model IDE should differ from a software IDE. One way relates to model visualization. It is common to visualize biochemical models as a directed graph. So, there is a requirement to have bidirectional visualization and editing of the text and graphical representation of a model. Beyond this, it is likely desirable to provide for the easy integration of many tools with the source editor, such as checking for stoichiometric inconsistencies (Gevorgyan *et al.*, 2008).

We further expect that debugging a biochemical model differs from debugging software. Debugging software is done by isolating where the error occurs. However, as Section 3.4 illustrates, there is often no convenient way to isolate problems with a biochemical model because of the interconnectedness of chemical species and reactions. Revisiting the scenario in Section 3.4, the concentration of Y is too small and the concentration of Yp is too large. Analyzing this problem requires analyzing the reactions that synthesize and/or degrade chemical species to understand why reaction fluxes are too large or too small. The explanation may be that some species in a reaction’s rate law is too large (small). This means that the analysis can be recursive, adding to the complexity of debugging biochemical models.

In the near term, we are addressing limitations of our current implementation. Specifically, we want to address support for SBML packages such as Flux Balance Constraints and statistical distributions (distrib).

## 5 Conclusion

Developing models in systems biology is a complex, knowledge-intensive activity. Drawing on the experience of software engineering, we believe that expert modelers can benefit from good tools for modeling using text-based representations of models. Herein, our focus is on Antimony, a human readable representation of SBML models.

At present, the tools for text-based model development are limited, typically just a textual editor that provides features such as copy, paste, find, and replace. This motivated the development of VSCode-Antimony, an editor that is model aware and so can provide sophisticated features for building, analyzing, and translating models written in the Antimony modeling language. For example, VSCode-Antimony provides autocompletion of variable names to assist with model building, hover messages that aid in model analysis, and translation between XML and Antimony representations of SBML models. These features result from making VSCode-Antimony model-aware by incorporating several sophisticated capabilities: analysis of the Antimony grammar (e.g. to identify model symbols and their types); a query system for accessing knowledge sources for chemical species and reactions; and automatic conversion between different model representations (e.g. between Antimony and SBML).

VSCode-Antimony is implemented as an extension to VSCode. It is freely available through the VSCode marketplace.
